# Occupational and Environmental Asbestos Exposure and Survival of Patients with Asbestos-Related Cancer: A Follow-Up Study on Patients with Malignant Mesothelioma and Asbestos-Related Lung Cancer in Korea

**DOI:** 10.3390/toxics12010020

**Published:** 2023-12-25

**Authors:** Min-Sung Kang, Woo-Ri Chae, Yong-Jin Lee, Kyong Whan Moon

**Affiliations:** 1 Asbestos Environmental Health Center, Soonchunhyang University Cheonan Hospital, Soonchunhyang 6-gil 31, Dongnam-gu, Cheonan-si 31151, Republic of Korea; kms83korea03@hanmail.net (M.-S.K.); lyj34274@daum.net (Y.-J.L.); 2 Department of Health and Safety Convergence Science, Korea University, Anam-ro 145, Seongbuk-gu, Seoul 02841, Republic of Korea; wr245@korea.ac.kr; 3 Department of Occupational & Environmental Medicine, Soonchunhyang University, Soonchunhyang 6-gil 31, Dongnam-gu, Cheonan-si 31151, Republic of Korea; 4 School of Health and Environmental Science, Korea University, Anam-ro 145, Seongbuk-gu, Seoul 02841, Republic of Korea; 5 BK21 FOUR R&E Center for Learning Health System, Korea University, Anam-ro 145, Seongbuk-gu, Seoul 02841, Republic of Korea

**Keywords:** asbestos, lung cancer, malignant mesothelioma, survival

## Abstract

Malignant mesothelioma and asbestos-related lung cancer are typically associated with a poor prognosis. However, it has been observed that some patients with these cancers survive significantly longer than the average survival period. While many preliminary studies have investigated factors influencing patient survival, the specific impact of asbestos exposure has not been thoroughly explored. We followed up with 546 patients with malignant mesothelioma and 902 patients with asbestos-related lung cancer, all identified as asbestos victims between 2009 and 2021. In both malignant mesothelioma and asbestos-related lung cancer, patients with occupational asbestos exposure exhibited not only shorter median survival times but also lower 3- and 5-year survival rates compared to those with environmental exposure. Additionally, a longer duration of occupational exposure and closer proximity to the source of asbestos were linked to shorter survival times and lower survival rates. Among the patients with occupational asbestos exposure, the highest hazard ratios (HRs) were observed in those who worked in the production of asbestos-containing products across both cancer types. In contrast, significant HRs were only noted in mesothelioma patients who lived near asbestos industries, slate houses, and redevelopment areas, within the environmentally exposed group.

## 1. Introduction

Asbestos was considered an essential mineral for many post-industrial activities due to its heat resistance, durability, insulating properties, warmth, and low cost [1]. However, most countries have banned asbestos because of the health problems it causes [2]. It is now well established that both malignant mesothelioma and asbestos-related lung cancer are associated with asbestos exposure [3]. In 2019, an estimated 35,000 malignant mesothelioma patients died worldwide [4], and approximately 180,000 lung cancer deaths each year are attributed to asbestos exposure [5].

Malignant mesothelioma and asbestos-related lung cancer generally have a poor prognosis, with malignant mesothelioma, in particular, having a five-year survival rate of less than 5% [6]. However, it has been recognized that some patients with asbestos-related cancers can survive significantly longer than the average [7,8]. Previous studies have re-ported that demographic characteristics (such as gender, age, race, and socioeconomic level) [9,10,11,12,13], lifestyle behaviors (like smoking) [12,14], and specific genetic factors [9,15] are associated with survival in patients with asbestos-related cancers. Identifying which factors are associated with a patient’s survival is important to determine patients at high risk of death. To this end, Linton et al. developed a risk score to identify patients who are likely to have a shorter survival time [16].

Another factor associated with survival in patients with asbestos-related cancers is the characteristic of asbestos exposure. Recent studies have suggested that characteristics of asbestos exposure may influence the survival of patients with malignant mesothelioma and asbestos-related lung cancer. Gao et al. analyzed data from 748 patients in the US Na-tional Mesothelioma Virtual Bank and found that among industries where malignant mesothelioma was documented, manufacturing and construction were the most frequent [10]. Noelle et al. analyzed data from 702 lung cancer patients at the Comprehensive Cancer Center Leon Berard in Lyon, France, and reported that the survival of lung cancer patients occupationally exposed to asbestos might be shorter compared to that of those not exposed [17]. However, these studies only examined the presence of occupational asbestos exposure or the type of occupation without considering factors such as the duration and frequency of exposure or the presence of environmental asbestos exposure. The potential impact of asbestos on malignant mesothelioma and asbestos-related lung cancer remains under-investigated [17].

Therefore, the aim of this study was to investigate the impact of asbestos exposure characteristics on the survival of patients with asbestos-related cancers. We examined the type, duration, and frequency of asbestos exposure based on the asbestos exposure histories of patients with malignant mesothelioma and asbestos-related lung cancer. These patients were then followed to estimate the impact of each factor on their survival.

## 2. Materials and Methods

### 2.1. Study Population

We utilized data on individuals exposed to asbestos gathered by the South Korea Ministry of Environment (MOE). According to the Asbestos Injury Relief Act, the MOE is mandated to continually collect data from individuals affected by asbestos and assess if their injuries are attributable to asbestos exposure. The MOE created the Environmental Health Center for Asbestos in 2009, which conducts health surveys on local residents suspected of asbestos exposure [18]. Additionally, data on individuals impacted by asbestos are gathered via regular medical examinations. In South Korea, the National Health Insurance Act mandates adults to undergo routine medical check-ups, with healthcare facilities required to report any suspected cases of asbestos-related harm to the MOE. Our study analyzed data obtained through this framework, focusing on information from 546 patients with malignant mesothelioma and 902 patients with asbestos-related lung cancer, all identified as asbestos victims from 2009 to 2021.

### 2.2. History of Asbestos Exposure

The Environmental Health Center for Asbestos developed a structured questionnaire to investigate the asbestos exposure history of the patients. The survey was conducted by researchers involved in the development of the questionnaire. Asbestos exposure was categorized into occupational and environmental exposure. Co-exposure was considered an occupational exposure in our analysis because occupational asbestos exposure levels are generally higher than environmental exposure levels.

Occupational asbestos exposure was defined as occupational exposure to asbestos fibers in workplace. The survey items for occupational exposure included the name of the workplace, type of job, work duration, and age at first exposure. Job types were classified into five categories: extraction work (involving extraction, conveyance, and grinding), production of asbestos-containing products (such as cement, slate, and fabric), construction (involving construction or demolition of asbestos-containing buildings), maintenance work (repairing asbestos-containing buildings or equipment), and others. To minimize information bias from response inaccuracies, we verified employment certificates and past records confirming business locations and operation periods.

Environmental asbestos exposure was defined as non-occupational exposure to airborne asbestos fibers caused by sources such as asbestos mines and factories. Survey items for environmental exposure included the area of residence, type of exposure source, distance from the source, residence duration, and age at first exposure. The types of exposure source were classified into six categories: asbestos mine, asbestos industry, shipyard, slate house, redevelopment area, and others. To ensure the accuracy of the exposure information, we checked resident registration documents of the patients and historical records of the exposure sources’ locations and operation periods.

### 2.3. Survival Outcome

Cancer-specific survival time was measured from the cancer diagnosis date to the date of death caused by malignant mesothelioma (as per the Korean standard classification of diseases [KCD]-8 code C45) or lung cancer (KCD-8 code C34). For patients who were alive at the last follow-up date, their survival duration was considered as the period from the date of their cancer diagnosis to 31 December 2021, which was the final date of follow-up. The number of patients who survived to the final date of the study was 154 for malignant mesothelioma and 572 for lung cancer.

### 2.4. Statistical Analysis

According to the characteristics of asbestos exposure, median survival duration and 3- and 5-year survival rates were calculated for patients with malignant mesothelioma and lung cancer.

Cox proportional hazard models were used to estimate the association of asbestos exposure with cancer-specific mortalities of patients while adjusting for potential confounders. These included sex (male and female), age at diagnosis (continuous), smoking status (never smoker, past smoker, current smoker, and unknown), cancer cell type for malignant mesothelioma (epithelioid, sarcomatoid, and biphasic) and lung cancer (adenocarcinoma, squamous cell, small cell, large cell, and others), and type of treatment (surgery, radiotherapy, and chemotherapy). The results were presented as hazard ratios (HRs) and 95% confidence intervals (CIs) for mortality.

All statistical analyses were performed using R version 4.3.0 [19], and a statistical significance level was set as a two-sided *p*-value < 0.05.

## 3. Results

Descriptive statistics and characteristics of patients in the study are presented in Table 1. The median survival times for patients with malignant mesothelioma and asbestos-related lung cancer were 1.58 and 2.92 years, respectively. More than half of the patients were men, and the mean ages at diagnosis were 63.1 and 65.3 years, respectively. Patients with malignant mesothelioma were predominantly exposed to environmental asbestos (48.2%), whereas lung cancer patients mainly experienced co-exposure to asbestos (43.3%).

The median survival duration and 3- and 5-year survival rates based on the occupational asbestos exposure are presented in Table 2. For both malignant mesothelioma and lung cancer, patients with occupational exposure to asbestos had shorter median survival times than those with environmental exposure, and this trend was also observed for 3- and 5-year survival rate. Survival time and survival rate declined with an increase in work duration. For malignant mesothelioma patients, the median survival time was 1.75 years for those who worked less than one year, which reduced to 1.00 year for those with a work history of more than 30 years. Similarly, in lung cancer patients, the median survival was 4.08 years for patients working less than a year, decreasing to 2.46 years for those with over 30 years of work experience. The association between survival and occupational status was inconsistent across the two cancer types.

The median survival duration and 3- and 5-year survival rates based on the environmental asbestos exposure are presented in Table 3. For both malignant mesothelioma and lung cancer, patients residing closer to the exposure source experienced shorter survival times and lower survival rates. For malignant mesothelioma patients, the median survival time was 2.42 years for those living more than 5 km away from the exposure source, decreasing to 1.25 years for those living less than 0.5 km away. Similarly, in lung cancer patients, the median survival time was 4.67 years for those living more than 5 km away from the exposure source, decreasing to 2.17 years for those living less than 0.5 km away. The association between survival and type of exposure source was inconsistent across the two cancer types.

Table 4 shows the results of applying Cox regression analysis to estimate the effect of occupational asbestos exposure on cancer-specific mortalities. In malignant mesothelioma, the HR for patients with occupational exposure to asbestos was 1.31 compared to that of those with environmental exposure after adjusting for covariates. Additionally, the HR rose significantly with a 10-year increase in the work duration (HR = 1.10 [95% CI: 1.00, 1.20]). However, these associations were not observed in patients with lung cancer. For both malignant mesothelioma and lung cancer, the highest HRs were observed in patients who worked in production of asbestos-containing products. For the remaining occupation types, a positive association with mortality was observed, but not statistically significant.

Table 5 shows the effect of environmental asbestos exposure on cancer-specific mortalities. The HRs rose significantly with a 10-year increase in the residence duration in patients with malignant mesothelioma (HR = 1.12 [95% CI: 1.04, 1.21]) and lung cancer (HR = 1.15 [95% CI: 1.05, 1.25]). In addition, the distances from the source were negatively associated with HR in patients with malignant mesothelioma (HR = 0.87 [95% CI: 0.78, 0.97]) and lung cancer (HR = 0.86 [95% CI: 0.73, 1.01]). For mesothelioma, significant HRs were observed in patients who lived near asbestos industries (HR = 2.17 [95% CI: 1.16, 4.03]), slate houses (HR = 2.22 [95% CI: 1.04, 4.75]), and redevelopment areas (HR = 2.10 [95% CI: 1.10, 4.01]). In lung cancer, relatively large HRs were observed for asbestos industries (HR = 1.23 [95% CI: 0.37, 4.06]) and redevelopment sites (HR = 1.34 [95% CI: 0.26, 6.80]), but these were not statistically significant.

## 4. Discussion

In this study, we investigated the effects of occupational and environmental asbestos exposure on the survival of patients with mesothelioma and asbestos-related lung cancer. In both malignant mesothelioma and asbestos-related lung cancer, patients with occupational asbestos exposure exhibited not only shorter median survival times but also lower 3- and 5-year survival rates compared to those with environmental exposure. Additionally, a longer duration of occupational exposure and closer proximity to the exposure source were associated with shorter survival times and lower survival rates. Among patients who had occupational asbestos exposure, the highest HRs were noted in those who had worked in the production of asbestos-containing products for both types of cancer. In contrast, significant HRs were only observed in mesothelioma patients who had lived near asbestos industries, slate houses, and redevelopment areas among those with environmental exposure.

South Korea produced or imported approximately 2 to 2.4 million tons of asbestos from the time it began using asbestos until its ban in 2009 [18]. Due to the long latency period of asbestos-related diseases, cases of mesothelioma and lung cancer continue to occur to this day. In 2011, the Korean Ministry of Environment enacted the Asbestos Injury Relief Act and established the Environmental Health Center for Asbestos [20]. The primary role of this center is to operate an asbestos health surveillance system, conducting health impact assessments in areas with suspected asbestos exposure [18]. Asbestos exposure characteristics differ regionally; for example, about 60% of the asbestos mines in Korea are located in Chungcheongnam-do, resulting in many patients in this region having worked in mining or lived near asbestos mines [21]. This study presents results based on the types of occupational and environmental sources of asbestos exposure, which can aid in prioritizing areas for further investigation by asbestos environmental health centers and in predicting the risk for asbestos victims in specific regions.

The relationship between asbestos exposure and the survival of patients with asbestos-related cancers has been explored in several previous studies, but the results have been inconsistent. Studies conducted by Flores et al. [22], Gao et al. [10], and Noelle et al. [17] indicated decreased survival in patients with cancers exposed to asbestos compared to those unexposed. In contrast, studies by Berardi et al. [23], Nojiri et al. [24], and Gorini et al. [25] found no significant association between asbestos exposure and patient survival in asbestos-related cancer cases. However, considering the relatively large number of subjects in studies that did find an association (over 700), this inconsistency may stem from statistical power limitations. The studies carried out by Berardi et al. [23], Nojiri et al. [24], and Gorini et al. [25], which did not observe an association, included 62, 314, and 381 patients, respectively. Another limitation in the existing research is the approach to investigating asbestos exposure history. Most of the previous studies focused solely on occupational asbestos exposure, with only one exploring the specific occupational settings of exposure [10]. Conversely, our study indicates that not just occupational asbestos exposure, but also the duration of work and proximity to the exposure source, can influence patient survival. Thus, our findings provide more substantial evidence that exposure to asbestos may decrease the survival of patients with mesothelioma and asbestos-related lung cancer.

It is noteworthy that HRs were observed to be relatively higher in patients who worked in the production of asbestos-containing products and those living near asbestos factories, compared to other exposure sources. Although there is no biological hypothesis to fully explain this, one plausible explanation could be the differences in the types of asbestos to which they were exposed. Various types of asbestos have differing levels of harm, and it is widely recognized that crocidolite and amosite are more harmful than chrysotile, commonly known as white asbestos [26]. In Korea, while most of the domestically produced asbestos is chrysotile, crocidolite and amosite were imported and utilized in specific industries, such as in the production of asbestos-containing products [27]. Records from the South Korea Ministry of Employment and Labor indicate that the use of crocidolite and amosite was relatively higher in factories producing asbestos-containing products compared to that in other industries [28]. A prior study reported that 43% of workers in these asbestos factories were exposed to crocidolite [29]. Consequently, it is feasible that patients who worked in or lived near asbestos factories were exposed to more harmful types of asbestos, potentially leading to shorter survival times. This finding is similar to that of a previous study investigating the latency period of asbestos-related diseases by occupation type. In a previous study analyzing the latency period of asbestos-related diseases, patients who worked in the production of asbestos-containing products or lived near asbestos factories experienced a shorter latency period before developing mesothelioma and lung cancer compared to those exposed to other sources of asbestos [30].

Recent research, such as a study on the acute toxicity of asbestos fibers, has reported the distinct carcinogenic mechanisms of different types of asbestos [31]. Crocidolite, known for its high biodurability, promotes carcinogenesis through persistent cellular interactions, leading to DNA damage and chronic inflammation due to its ability to generate reactive oxygen species. In contrast, chrysotile, characterized by lower biodurability, causes cellular harm through the release of toxic metals and ROS production, triggering similar pathways of DNA damage and inflammation. This divergence in the pathological pathways between crocidolite and chrysotile underscores the complexity of asbestos-induced carcinogenesis and suggests that different types of asbestos may have different effects on cancer development and survival from cancer.

Several limitations of the current study should be acknowledged. Firstly, due to a lack of data, asbestos fiber concentration levels were not available for both occupational and environmental exposures, leading to a limited assessment of exposure. Nevertheless, considering the scarcity of concrete asbestos exposure level data in many prior studies, surrogate indicators like exposure duration and proximity to exposure sources can serve as valid approaches for estimating exposure [32]. However, it is important to note that the count of asbestos bodies is a reliable indicator of asbestos exposure and should be considered in future research. Secondly, this study did not consider the educational and income levels of the patients. Although these variables were initially included in the questionnaire, they were subsequently omitted as most respondents were reluctant to disclose their educational and income levels. Future research should aim to collect comprehensive data to properly account for the participants’ socioeconomic status. Third, our study did not consider the genetic factors of the patients. The significance of mutations, such as BAP1, as a factor influencing susceptibility to asbestos-related diseases is gaining increasing recognition [33,34,35]. However, data pertaining to this aspect were not available in Korea. Should a national survey be conducted to explore potential associations between asbestos-related diseases and such mutations, these considerations could be included in future studies.

## 5. Conclusions

Our results offer more substantial evidence suggesting that asbestos exposure may reduce the survival times of patients with malignant mesothelioma and asbestos-related lung cancer. Despite the widespread ban on asbestos use in many countries, asbestos-related diseases continue to be a significant global public health issue due to their prolonged latency period. Consequently, further investigation into the patterns of asbestos exposure and the development of strategies to enhance the survival of individuals with asbestos-related diseases is imperative.

## Figures and Tables

**Table 1:** Descriptive statistics and characteristics of patients in this study.

Variables	Malignant Mesothelioma (n = 546)	Lung Cancer (n = 902)
Median survival duration (years)	1.58	2.92
Sex, n (%)		
Male	337 (61.7)	570 (63.2)
Female	209 (38.3)	332 (36.8)
Age at diagnosis, mean ± SD (years)	63.1 ± 12.9	65.3 ± 9.4
Year of diagnosis, n (%)		
2009–2012	132 (24.2)	65 (7.2)
2013–2015	128 (23.4)	147 (16.3)
2016–2018	128 (23.4)	288 (31.9)
2019–2021	158 (29.0)	402 (44.6)
Smoking status, n (%)		
Never smoker	270 (49.5)	407 (45.1)
Past smoker	238 (43.6)	430 (47.7)
Current smoker	6 (1.1)	7 (0.8)
Unknown	32 (5.9)	58 (6.4)
Exposure modalities, n (%)		
Occupational	214 (39.2)	133 (14.8)
Environmental	263 (48.2)	378 (41.9)
Co-exposure	59 (12.6)	391 (43.3)
Treatment types (multiple responses), n (%)		
Surgery	161 (29.5)	430 (47.7)
Radiotherapy	9 (1.6)	126 (14.0)
Chemotherapy	284 (52.0)	371 (41.1)

**Table 2:** Median survival duration and 3- and 5-year survival rates based on the occupational asbestos exposure.

Variables	Malignant Mesothelioma				Lung Cancer			
	n	Median Survival (Years)	Survival Rate (%)		n	Median Survival (Years)	Survival Rate (%)	
			3-Year	5-Year			3-Year	5-Year
Asbestos exposure modalities								
Environmental exposure	263	1.75	47.5	39.2	378	3.04	77.8	70.6
Occupational exposure	286	1.33	33.6	23.7	524	2.92	72.3	66.0
Age at first occupational exposure (years)								
<20	56	1.67	33.6	31.1	137	3.33	86.1	82.6
20–29	93	0.83	24.1	21.3	153	2.42	77.3	70.6
30–39	74	1.33	37.4	36.6	125	3.17	56.9	47.7
40–49	37	2.08	49.3	37.5	68	3.54	94.3	86.8
≥50	26	1.58	38.1	28.6	41	3.00	74.6	68.0
Work duration (years)								
<1	15	1.75	38.5	28.2	58	4.08	85.9	71.9
1–5	44	1.33	50.0	40.0	89	2.50	81.8	75.8
5–10	40	1.50	37.6	25.6	90	2.67	68.9	64.0
10–30	121	0.92	17.1	14.3	186	2.75	76.9	73.8
≥30	66	1.00	29.0	22.6	101	2.46	62.2	55.1
Types of job								
Others	70	2.08	52.9	38.6	80	2.42	72.4	69.0
Extraction ^1^	43	0.92	40.0	20.0	68	2.38	53.8	47.5
Production ^2^	31	1.08	50.0	35.0	222	2.79	80.9	79.4
Construction ^3^	109	1.25	24.8	16.4	67	3.33	75.2	67.6
Maintenance ^4^	33	1.25	26.1	21.7	87	3.42	76.1	65.7

^1^ Extraction refers to the process of extracting, conveying, and grinding asbestos fibers. ^2^ Production refers to the production of asbestos-containing products such as cement, slate, and fabric. ^3^ Construction refers to the process of construction or demolition of asbestos-containing buildings. ^4^ Maintenance refers to the process of repairing asbestos-containing buildings or equipment.

**Table 3:** Median survival duration and 3- and 5-year survival rates based on the environmental asbestos exposure.

Variables	Malignant Mesothelioma				Lung Cancer			
	n	Median Survival (Years)	Survival Rate (%)		n	Median Survival (Years)	Survival Rate (%)	
			3-Year	5-Year			3-Year	5-Year
Age at first environmental exposure (years)								
<20	87	1.83	39.0	28.6	124	3.33	77.7	69.8
20–29	60	1.50	30.5	21.3	81	3.33	91.2	82.4
30–39	62	1.75	52.2	49.3	99	2.33	90.7	88.0
40–49	29	1.42	40.3	31.1	47	2.50	58.8	48.5
≥50	25	1.67	48.1	37.4	27	2.67	77.8	70.6
Distance from the source (km)								
>5	9	2.42	50.0	44.7	11	4.67	72.7	72.7
2–5	71	2.25	53.8	47.7	120	3.08	73.3	65.0
1–2	68	1.79	46.0	38.1	118	3.58	80.5	74.6
0.5–1	34	1.88	39.3	25.0	51	2.67	80.8	73.1
≤0.5	81	1.25	50.0	37.5	78	2.17	78.4	70.6
Types of exposure source								
Others	45	1.00	60.0	60.0	13	2.50	84.6	84.6
Asbestos mine	33	1.17	21.7	21.7	82	3.17	54.2	45.8
Asbestos industry	88	1.75	47.7	36.7	150	3.38	78.9	71.1
Shipyard	35	2.00	60.0	52.0	76	2.88	92.4	86.4
Slate house	30	2.17	57.8	48.9	50	2.54	87.5	80.0
Redevelopment area	32	1.25	27.0	18.2	7	2.50	85.7	85.7

**Table 4:** Adjusted ^1^ hazard ratios (HRs) and 95% confidence intervals (CIs) for cancer-specific mortality associated with characteristics of occupational asbestos exposure.

Variables	Malignant Mesothelioma		Lung Cancer	
	HR (95% CI)	*p*-Value	HR (95% CI)	*p*-Value
Asbestos exposure modalities				
Environmental exposure	Ref		Ref	
Occupational exposure	1.31 (1.06, 1.62)	0.013	0.95 (0.75, 1.20)	0.669
Age at first exposure (per 1 year increase)	1.08 (0.97, 1.20)	0.156	1.04 (0.95, 1.12)	0.350
Work duration (per 10 years increase)	1.10 (1.00, 1.20)	0.040	1.01 (0.93, 1.09)	0.806
Types of job				
Others	Ref		Ref	
Extraction ^2^	1.69 (0.96, 2.66)	0.044	1.24 (0.78, 1.98)	0.365
Production ^3^	2.22 (1.06, 6.19)	0.077	1.92 (1.18, 3.11)	0.008
Construction ^4^	1.70 (0.89, 3.24)	0.107	1.39 (0.76, 2.53)	0.283
Maintenance ^5^	1.50 (0.84, 2.67)	0.169	1.30 (0.73, 2.32)	0.374

^1^ Models were adjusted for sex, age at diagnosis, smoking status, cancer cell type, and type of treatment. ^2^ Extraction refers to the process of extracting, conveying, and grinding asbestos fibers. ^3^ Production refers to the production of asbestos-containing products such as cement, slate, and fabric. ^4^ Construction refers to the process of construction or demolition of asbestos-containing buildings. ^5^ Maintenance refers to the process of repairing asbestos-containing buildings or equipment.

**Table 5:** Adjusted ^1^ hazard ratios (HRs) and 95% confidence intervals (CIs) for cancer-specific mortality associated with characteristics of environmental asbestos exposure.

Variables	Malignant Mesothelioma		Lung Cancer	
	HR (95% CI)	*p*-Value	HR (95% CI)	*p*-Value
Age at first exposure (per 1 year increase)	1.01 (0.90, 1.11)	0.853	1.01 (0.92, 1.09)	0.818
Residence duration (per 10 years increase)	1.12 (1.04, 1.21)	0.003	1.15 (1.05, 1.25)	0.002
Distance from the source (per 1 km increase)	0.87 (0.78, 0.97)	0.012	0.86 (0.73, 1.01)	0.069
Types of exposure source				
Others	Ref.		Ref.	
Asbestos mine	1.51 (0.95, 2.41)	0.083	1.17 (0.34, 4.03)	0.803
Asbestos industry	2.17 (1.16, 4.03)	0.015	1.23 (0.37, 4.06)	0.735
Shipyard	1.29 (0.63, 2.64)	0.486	0.81 (0.22, 2.97)	0.751
Slate house	2.22 (1.04, 4.75)	0.040	0.87 (0.22, 3.43)	0.843
Redevelopment area	2.10 (1.10, 4.01)	0.025	1.34 (0.26, 6.80)	0.725

^1^ Models were adjusted for sex, age at diagnosis, smoking status, cancer cell type, and type of treatment.

## Data Availability

The data presented in this study are available upon request from the corresponding author. The data are not publicly available because they contain sensitive patient information and location data.
